# Higher skeletal muscle mass is associated with higher blood pressure and left ventricular mass

**DOI:** 10.1097/HJH.0000000000004375

**Published:** 2026-06-20

**Authors:** Eduard Shantsila, Alena Shantsila, Matthew Sperrin, Zhuangzhi Gao, Yalin Zheng, Ian B. Wilkinson, Paul Leeson, Iain Buchan, Gregory Y.H. Lip

**Affiliations:** a Department of Primary Care and Mental Health; b Liverpool Centre of Cardiovascular Sciences; c Department of Cardiovascular and Metabolic Medicine, University of Liverpool; d Division of Informatics, Imaging & Data Science, University of Manchester; e Department of Eye and Vision Sciences, University of Liverpool; f Division of Experimental Medicine, University of Cambridge; g Addenbrooke's Hospital, Cambridge; h Division of Cardiovascular Medicine, University of Oxford; i John Radcliffe Hospital, Oxford; j Department of Public Health, Policy and Systems, Institute of Population Health, University of Liverpool, Liverpool, UK

**Keywords:** blood pressure, hypertension, skeletal muscles

## Abstract

**Background and aims:**

Skeletal muscles make up about 40% of the body mass, but the effects of skeletal muscle mass (SMM) on blood pressure (BP) and the heart remain unclear and were explored in this study.

**Methods:**

The study used cross-sectional and longitudinal cohort designs with data from 492 498 UK Biobank (ID 54078) participants without a history of stroke [mean (SD) age 56.5 (8.1) years, 45% men, 94% white]. Muscle mass index (MMI) was measured by bioimpedance analysis, lean mass index by DEXA, and thigh MMI by MRI, fat mass index (FMI) by bioimpedance and DEXA, left ventricular mass index (LVMI) by MRI. Multivariable regression was used to relate muscle mass, BP, and LVMI.

**Results:**

On cross-sectional analysis, people with hypertension have higher values of all muscle mass metrics, which were independently associated with higher SBP and DBP after adjustment for age, sex, use of BP-lowering drugs and their interactions. Each 1 kg/m^2^ higher muscle mass was associated with higher SBP by 0.67 [95% confidence interval (CI) 0.63–0.71] mmHg for MMI, 0.82 (95% CI 0.68–0.97) mmHg for lean mass index and 4.7 (95% CI 4.3–5.1) mmHg for thigh MMI. On longitudinal cohort analyses, higher values of muscle mass changes were independently associated with higher SBP and DBP after adjustment for age, sex and FMI (for SBP per 1 kg/m^2^ increase) MMI during first follow-up of 4.3 years was independently associated with 2.2 (95% CI 1.9–2.5) mmHg increase in SBP, with consistent findings during longer follow-ups and MMI assessment by DEXA and MRI (*P* < 0.001 for all). Similarly, higher values of muscle mass metrics and their follow-up changes were independently associated with higher LVMI on cross-sectional and cohort analysis.

**Conclusion:**

Higher SMM is associated with higher BP and LVMI, independently of adiposity. Further research is needed to establish whether the observed association is causal and to identify the optimal exercise modes for optimal BP and better health outcomes.

## INTRODUCTION

Studies show the beneficial effects of exercise on blood pressure (BP) [1]. Yet, it remains unclear what drives the benefits of physical activity, which activity is most beneficial for health and why not all physical activity is beneficial. Endurance, speed, strength and muscle mass are different outcomes of exercise influenced by exercise type and may differentially affect BP. Physical activity is inversely related to the incidence of cardiovascular disease and mortality, but the association is not linear, and the risk of coronary atherosclerosis is increased in athletes [1]. In fact, in middle-aged and older athletes, exercise intensity was related to the progression of coronary atherosclerosis [2].

Skeletal muscle accounts for approximately 40% of total body mass, yet it is seldom considered in clinical practice. Exercise, especially strength training, may be associated with increased skeletal muscle mass (SMM), which is often a desirable change and is often associated with a positive body image. It would be natural to expect that an increase in SMM would improve BP, but the scarce data on the topic come from small studies with conflicting results [3,4]. This study used measures of SMM obtained using different modalities [BIA, dual-energy X-ray absorptiometry (DEXA) and MRI) in the large prospective dataset of the UK Biobank to assess its relationship to BP and left ventricular mass, a surrogate measure of the cardiac effect of increased BP. While there are no methods to directly measure SMM *in vivo* in humans, these three methods based on different physics-biological principles are well validated for SMM estimation in adults. Their joint use in the study provides complementary evidence supporting the findings and reducing the risk of methodology-related bias if depending on a single methodology only.

## METHODS

### Study design and data sources

The study used cross-sectional and longitudinal cohort designs to explore associations between metrics of SMM and BP and LVMI. The analysis is based on UK Biobank data (UKBB ID 54078). The UK Biobank recruited 502 664 participants aged 40–70 years between 2006 and 2010 (response rate 5.5%) living within 25 miles of one of the 22 assessment centres in England, Scotland, and Wales. Study visits involved an initial assessment visit in 2006–2010, and three follow-up visits at 2012–2013 (follow-up visit 1), 2014+ (follow-up visit 2) and 2019+ (follow-up visit 3). DEXA and MRI scans were done during follow-up visits 2 and 3.

**FIGURE 1:**
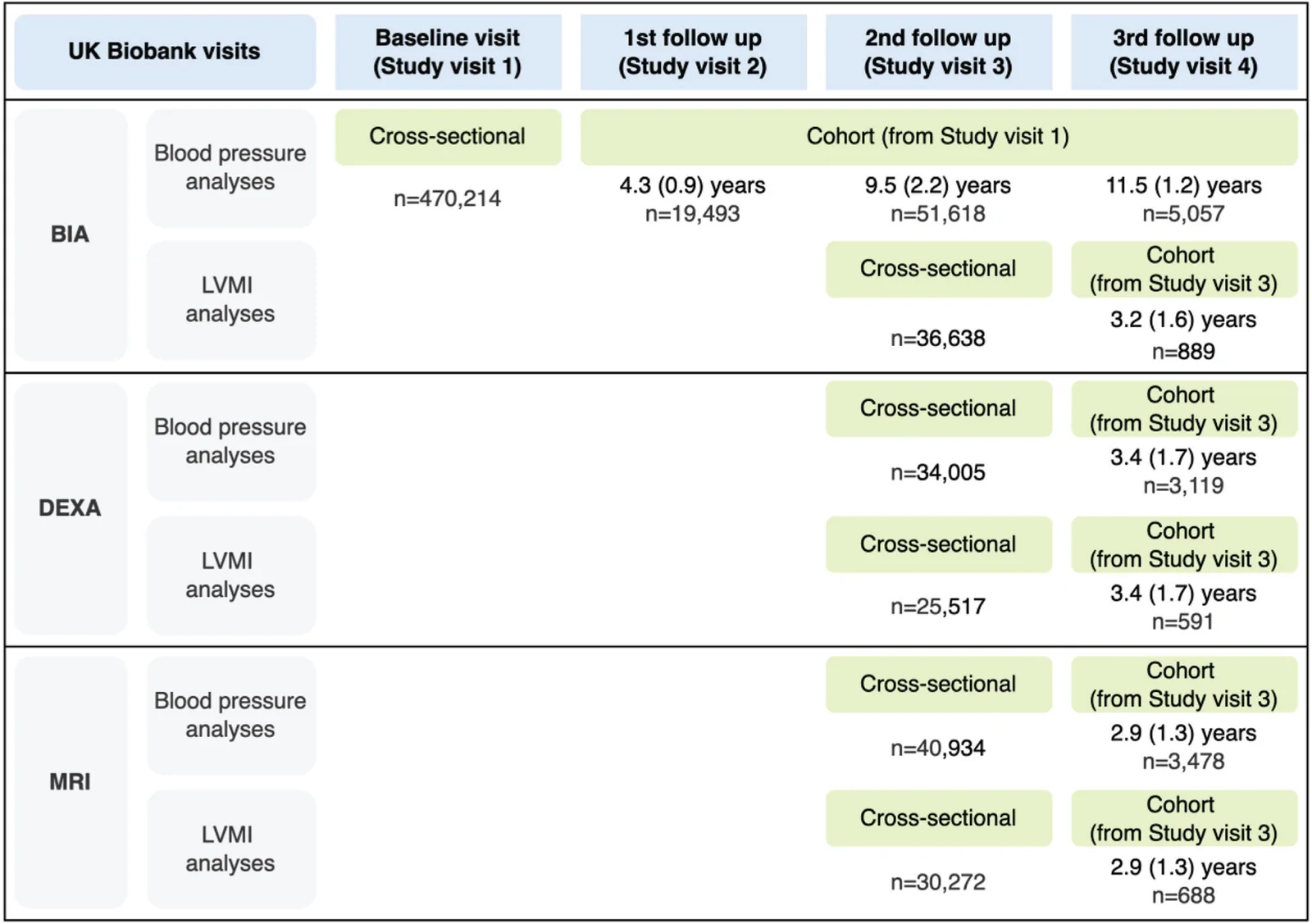
Study flow diagram. Numbers of participants in individual analyses could be lower due to missing values, exact numbers are provided in study tables. BIA, bioimpedance analysis; DEXA, dual-energy X-ray absorptiometry; LVMI, left ventricular mass index.

The cross-sectional analysis reflects inherited body phenotype and the influence of lifestyle. The cross-sectional analysis tests the associations at the point of initial assessment of muscle mass metrics using BIA (baseline visit), DEXA (follow up visit 2) and MRI (follow up visit 2) and corresponding BP and LVMI values. Cohort analysis captures the longitudinal changes in muscle mass dominated by lifestyle factors and their BP effects. The cohort analyses are based on prospectively collected data during the three BIA follow-ups for BP associations and one follow-up for LVMI association; one follow-up for DEXA and MRI for both BP and LVMI (Fig. 1). Cohort analyses included only participants completing both baseline and follow-up assessments and benefited from the predefined standardized follow-up duration. The analysis excludes patients with a history of stroke, due to its effects on mobility and muscle wasting with unknown individual impact. To explore age-related differences in BIA body composition and BP into more advanced age follow-up measurements were included in the descriptive presentation of the trend.

### Assessment of skeletal muscle mass

SMM was assessed using three methods: BIA, DEXA and MRI. The muscle mass metrics obtained using these methods are not fully interchangeable, but triangulation across them mitigates biases of individual methods, making conclusions more robust.

### Bioimpedance analysis

Bioimpedance analysis (BIA, Tanita BC418MA body composition analyser integrated into a weighing scale, TANITA Corporation, Tokyo, Japan) produced segmental values of fat mass, fat free mass and predicted muscle mass. Muscle mass index (MMI) was calculated as a sum of muscle mass of both arms, both legs and the trunk, divided by height squared. Fat mass index (FMI) was calculated as whole-body fat mass divided by height squared. BMI was calculated as body mass divided by height squared.

### Dual-energy X-ray absorptiometry

Full-body DEXA (iDXA, General Electric Lunar, Madison, Wisconsin, USA) was used to calculate whole body FMI (FMI-DEXA, whole body fat mass divided by height squared) and lean MMI (LMI, lean muscle mass divided by height squared) [5,6].

### MRI

MRI (Siemens 1.5 Tesla MAGNETOM Aera scanner, Siemens Healthineers, Erlangen, Germany with VD13A software) was used to calculate thigh MMI (TMMI) and left ventricular mass index (LVMI). Volumetric, co-aligned images of water and fat signal were acquired with a 6-min dual-echo Dixon Vibe protocol [6]. TMMI was calculated by multiplying total thigh muscle fat-free muscle volume by muscle density of 1.04 g/cm^3^, and converted to kg/m^2^[7]. LVM was measured using cardiac MRI. MRI did not provide a comparable measure of thigh fat mass. LVMI was calculated by division of left ventricular mass by body surface area.

All SMM metrics were normalized for height-squared (kg/m^2^). This approach allows for the appreciation of the comparability of SMM effects for BP and LVH across different modalities and for a more straightforward interpretation of the findings by researchers, clinicians and the public who are familiar with such normalization for body weight. It must be noted that while BIA and DEXA assess whole body SMM and are comparable in the effect size, MRI only analysed the mass of thigh muscle. While a major muscle group, whose mass also depends on height, it only represents a fraction of the overall muscle mass. A smaller absolute value of thigh muscle mass represents a relatively great change in overall SMM. In this study, the thigh muscle mass accounted for 20% of total SMM measured by BIA. In this regard, a 1 kg/m^2^ change in thigh muscle mass is roughly reflective of a 5 kg/m^2^ change in the total SMM measured by BIA, which must be considered when interpreting the findings.

### Blood pressure

Upper arm BP was measured after 5 min of seated rest using an appropriately sized cuff with an Omron 705 IT electronic BP monitor (Omron Healthcare, Kyoto, Japan). BP was measured twice at 1 min intervals. Manual measurements were made when automatic measurements could not be obtained. The mean of the two readings was used for this analysis.

### Statistical analysis

Continuous variables are summarized as mean (standard deviation, SD) for reasonably normally distributed data and median (25th quartile–75th quartile) otherwise, with medians compared using a Wilcoxon/Mann–Whitney test. Categorical data are summarized as counts (%) and compared using a chi-square test. To increase the lifespan of the captured age differences from 70 years (age limiting of UK Biobank recruitment) to 80 years descriptive analyses included values from follow-up visits. Analyses of association of body composition metrics with BP and LVMI were done for the whole population, followed by sensitivity analysis of populations that excluded outliers to improve generalizability of the findings. The excluded outliers were defined as values equal or outside 3 SDs or 1.5 interquartile ranges. The associations were analysed using univariable and multivariable linear and nonlinear regression. Bayesian information criterion (BIC) was calculated for linear and nonlinear models. Multivariable BP-prediction models for cross-sectional analyses included muscle mass metric (MMI for BIA, LMI for DEXA, TMMI for MRI), and additional adjustments for age, sex, use of BP-lowering drugs, FMI (BIA and DEXA) and their interactions with the muscle mass metric. Multivariate cross-sectional analyses of association of muscle mass metrics with LVMI included age, sex and FMI (BIA and DEXA). Longitudinal cohort (follow-up) analyses tested the association between changes in skeletal mass indices and changes in BP and LVMI. The changes were calculated as differences in their values between the baseline and the specified follow-up time points. Multivariable longitudinal models were adjusted for age, sex and FMI (BIA and DEXA). All analyses were done using records that had data for all variables included in the models (i.e. no missing data). For follow-up analyses, cohorts with matching baseline and follow-up records were used. The number of participants included in each analysis is provided in the corresponding tables. Multicollinearity was assessed using the variance inflation factor (VIF, car module, vif function). As all variables in the study models were below the threshold of 5, no additional handling was needed—the specified approach in the Statistical analysis section. Analyses were done using R version 4.3.1 with R Markdown 2.25.

## RESULTS

### Population

The full dataset included 502 389 participants. After excluding 7667 participants with a history of stroke and a further 2224 without information on stroke, 492 498 participants were included [age 56.5 (8.1) years, 45% men, 94% white]. Hypertension was present in 131 476 (27%) of participants. BP-lowering drugs were used at baseline by 91 135 (69%) patients with records of hypertension and 8085 (2%) without hypertension. Rates of raised BP in categories of hypertension and BP-lowering drug use status are shown in Supplement Table 1. Numbers and characteristics of participants for cross-sectional BIA, DEXA and MRI analyses are shown in Fig. 1, Table 1 (whole population) and Supplement Table 2 (sensitivity analysis). Characteristics of participants for follow-up BIA, DEXA and MRI analyses are shown in Table 2 (whole population) and Supplement Table 3 (sensitivity analysis).

**TABLE 1:** Baseline characteristics by methods of body composition assessment

Characteristic	Overall	No hypertension	Hypertension
BIA			
*n*	470 214	345 379	124 835
Age (years)	56 (8)	55 (8)	59 (7)^a^
Male sex [*n* (%)]	212 002 (45%)	148 495 (43%)	63 507 (51%)^a^
White ethnicity [*n* (%)]	445 948 (95%)	328 394 (95%)	117 554 (94%)^a^
Smoking [*n* (%)]	48 608 (10%)	37 033 (11%)	11 575 (9.3%)^a^
Diabetes [*n* (%)]	23 465 (5.0%)	8708 (2.5%)	14 757 (12%)^a^
Myocardial infarction [*n* (%)]	9772 (2.1%)	4735 (1.4%)	5037 (4.0%)^a^
BP-lowering medications [*n* (%)]	94 929 (20%)	7565 (2.2%)	87 364 (70%)^a^
Cholesterol-lowering medications [*n* (%)]	77 589 (17%)	30 860 (8.9%)	46 729 (37%)^a^
SBP (mmHg)	138 (19)	134 (18)	147 (18)^a^
DBP (mmHg)	82 (10)	81 (10)	86 (10)^a^
BMI (kg/m^2^)	27.4 (4.7)	26.7 (4.4)	29.4 (5.2)^a^
Muscle mass index (kg/m^2^)	17.7 (2.5)	17.4 (2.4)	18.4 (2.6)^a^
Fat mass index (kg/m^2^)	8.8 (3.6)	8.4 (3.4)	10.0 (4.0)^a^
DEXA			
*N*	34 005	27 097	6,908
Age (years)	64 (8)	63 (8)	67 (7)^a^
Male sex [*n* (%)]	16 424 (48%)	12 437 (46%)	3987 (58%)^a^
White ethnicity [*n* (%)]	32 859 (97%)	26 198 (97%)	6661 (96%)^b^
SBP (mmHg)	139 (19)	137 (19)	148 (18)^a^
DBP (mmHg)	79 (10)	78 (10)	82 (10)^a^
Lean mass index (kg/m^2^)	16.4 (2.1)	16.2 (2.1)	17.1 (2.2)^a^
Fat mass index (kg/m^2^)	9.1 (3.4)	8.8 (3.3)	10.2 (3.7)^a^
MRI			
*n*	40 934	32 758	8,176
Age (years)	64 (8)	63 (8)	67 (7)^a^
Male sex [*n* (%)]	19 577 (48%)	14 893 (45%)	4684 (57%)^a^
White ethnicity [*n* (%)]	39 710 (97%)	31 787 (97%)	7923 (97%)^b^
SBP (mmHg)	140 (19)	138 (19	148 (18)^a^
DBP (mmHg)	79 (10)	78 (10)	82 (10)^a^
Thigh muscle mass index (kg/m^2^)	3.6 (0.6)	3.6 (0.6)	3.7 (0.6)^a^
MRI Left ventricular mass index	*n* = 30 272		
Left ventricular mass index (g/m^2^)	45.7 (8.5)	45.1 (8.3)	48.2 (9.0)^a^

BP, blood pressure; BIA, bioimpedance analysis; DEXA, dual-energy X-ray absorptiometry.

a *P* < 0.001 vs. no hypertension.

b *P* > 0.05 vs. no hypertension.

**TABLE 2:** Metrics of body composition, blood pressure and left ventricular mass index during study visits

	Study visit 1	Study visit 2	Study visit 3	Study visit 4
BIA				
*n*	470 214	19 493	51 618	5057
Follow-up (years)	–	4.3 (0.9)	9.5 (2.2)	11.5 (1.2)
Age (years)	56 (8)	61 (7)	64 (8)	64 (7)
Males (%)	45%	49%	48%	48%
MMI (kg/m^2^)	17.5 (2.4)	17.4 (2.4)	17.3 (2.4)	17.1 (2.4)
FMI (kg/m^2^)	8.4 (3.0)	8.6 (3.5)	8.5 (3.4)	8.5 (3.3)
SBP (mmHg)	138 (19)	139 (18)	140 (19)	141 (19)
DBP (mmHg)	82 (10)	80 (10)	79 (10)	80 (10)
LVMI (*n*)			36 638	889
LVMI follow-up (years)			–	3.2 (1.6)
LVMI (g/m^2^)			45.6 (8.6)	46.0 (8.3)
DEXA				
*n*			34 005	3,119
Follow-up (years)				3.4 (1.7)
Age (years)			64 (8)	64 (7)
Males (%)			49%	49%
LMI (kg/m^2^)			16.4 (2.1)	16.3 (2.1)
FMI (kg/m^2^)			9.1 (3.4)	8.9 (3.5)
SBP (mmHg)			139 (19)	140 (19)
DBP (mmHg)			79 (10)	79 (10)
LVMI (*n*)			25 517	591
LVMI follow-up (years)			–	3.4 (1.7)
LVMI (g/m^2^)			45.8 (8.6)	46.0 (8.2)
MRI				
*n*			40 934	3478
Follow-up (years)				2.9 (1.3)
Age (years)			64 (8)	64 (7)
Males (%)			48%	49%
TMMI (kg/m^2^)			3.6 (0.6)	3.6 (0.6)
SBP (mmHg)			140 (19)	141 (19)
DBP (mmHg)			79 (10)	80 (10)
LVMI (*n*)			30 272	688
LVMI follow-up (years)				2.9 (1.3)
LVMI (g/m^2^)			45.7 (8.5)	46.0 (8.3)

BP, blood pressure; BIA, bioimpedance analysis; DEXA, dual-energy X-ray absorptiometry; LVMI, left ventricular mass index; MMI, muscle mass index; TMMI, thigh MMI.

### Age-related differences in body composition and blood pressure

**FIGURE 2:**
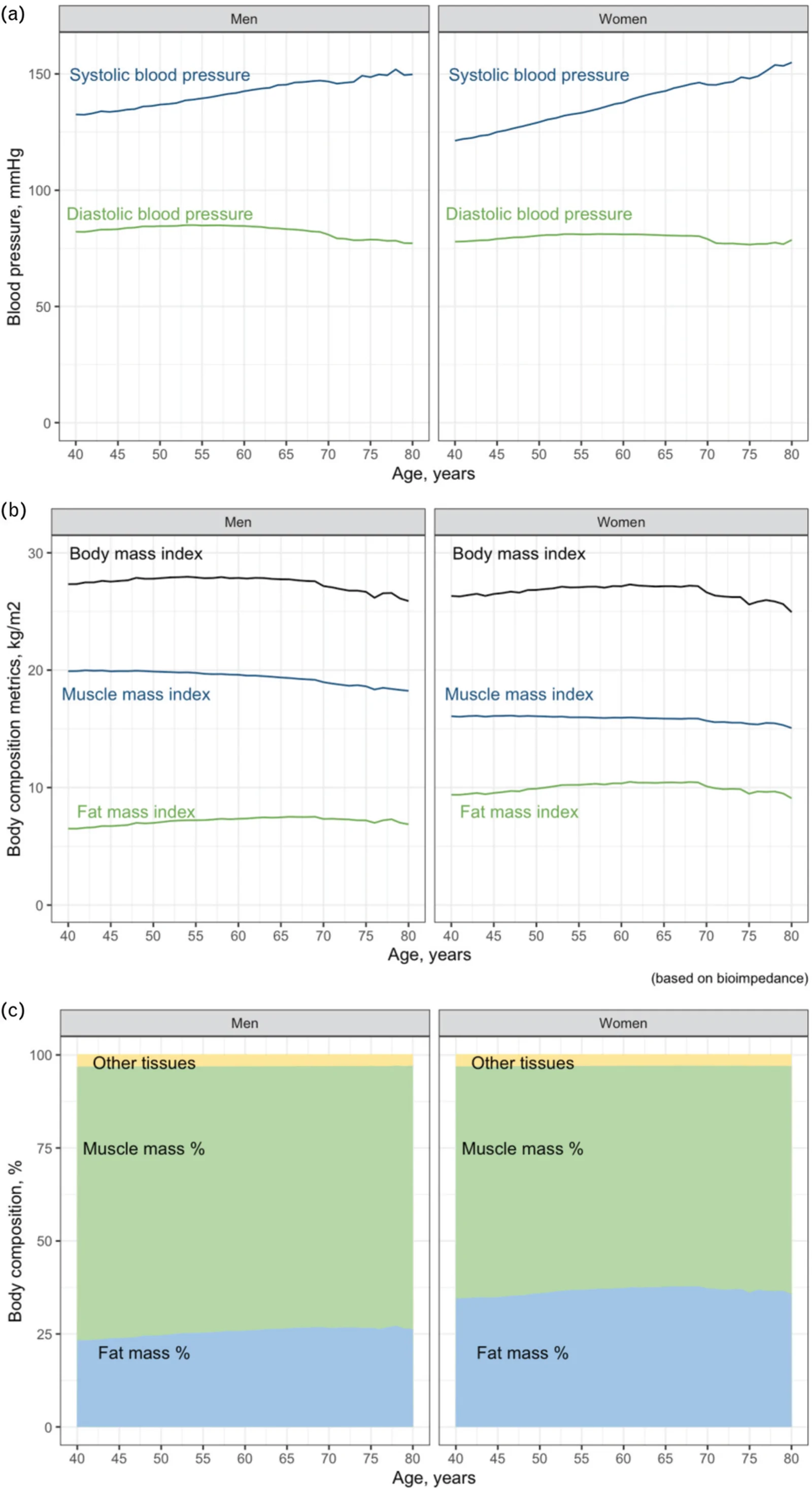
Age-related differences in body composition and blood pressure. (a) Changes in blood pressure. (b) Changes in body compositions (mass index, bioimpedance). (c) Changes in body compositions (%, bioimpedance). The body composition proportions reflect compartments measured by BIA.

Body composition and BP differed between individuals of different ages (Fig. 2, Supplement Table 4). Mean SBP increased across the age span from 133 (14) mmHg at age 40–44 years, to 150 (19) mmHg at age 75–80 years in men and 123 (15) mmHg to 151 (21) mmHg in women. Mean DBP reduced starting from the age of 60 from 84 (10) mmHg to 78 (10) mmHg in men and from 81 (10) mmHg to 77 (11) mmHg in women. BMI peaked at 60–64 in men and 65–70 in women, with a downward trend afterwards, with comparable overall values in men and women. In contrast, men consistently had higher MMI and lower FMI than women. There was a gradual decline in MMI in both genders from age 50, which paralleled an increase in FMI about age of 70, followed by a decline. BMI did not reflect the bidirectional changes in MMI and FMI, as show in the figure of relative changes in body fat and skeletal muscles (Fig. 2).

### Muscle mass metrics in hypertension

In the cross-sectional analysis, participants with hypertension vs. no hypertension had higher MMI 18.4 (2.6) vs. 17.4 (2.4), LMI 17.1 (2.2) vs. 16.2 (2.1) and TMMI 3.7 (0.6) vs. 3.6 (0.6) (*P* < 0.001 for all, Table 1), with a similar pattern on sensitivity analysis (Supplement Table 2).

### SBP

**FIGURE 3:**
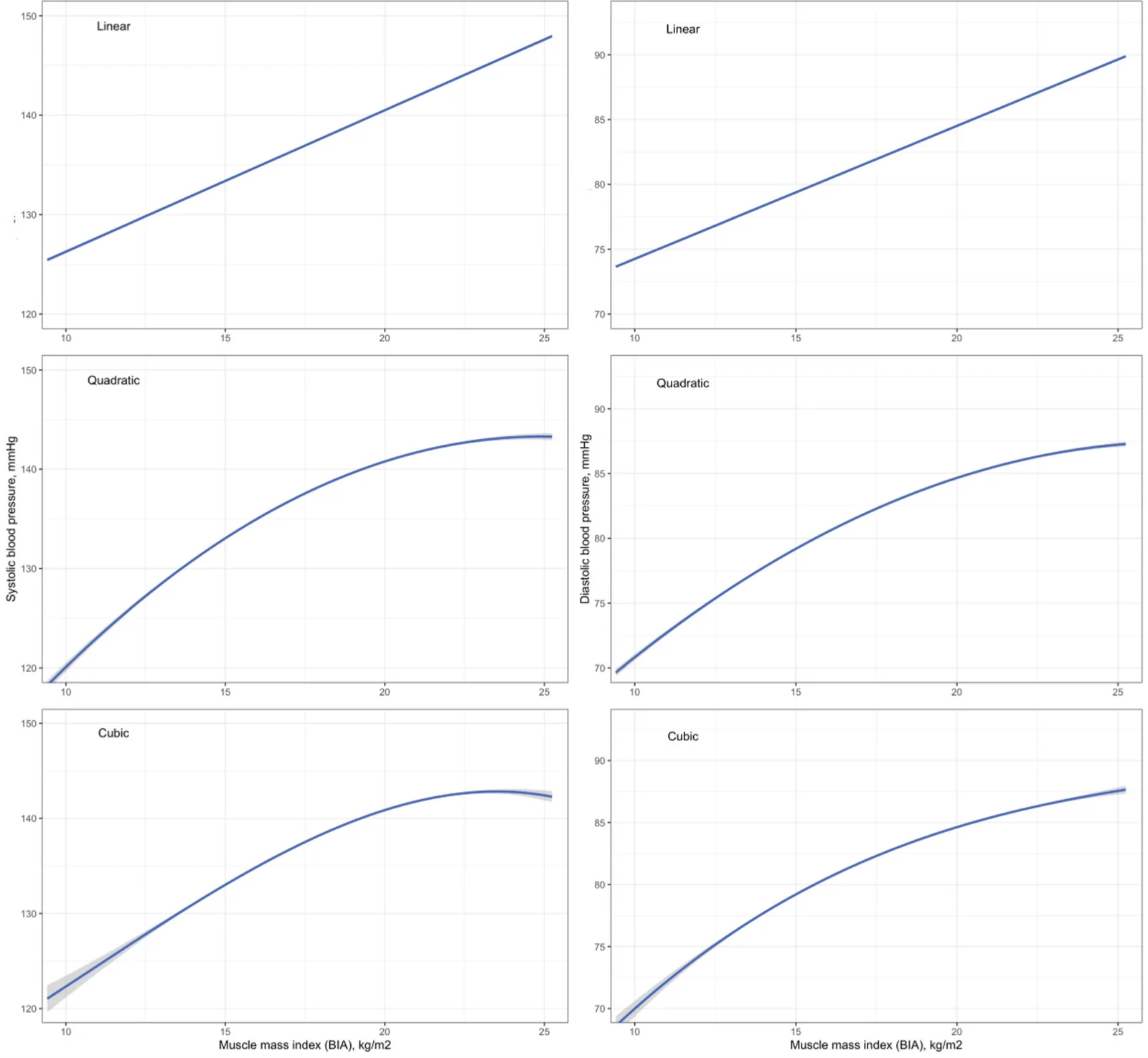
Relationship between bioimpedance muscle mass metrics and blood pressure (baseline cross-sectional data). The left panels show SBP effects, the right panels show DBP effects (linear, quadratic, and cubic). Based on values excluding outliers.

**FIGURE 4:**
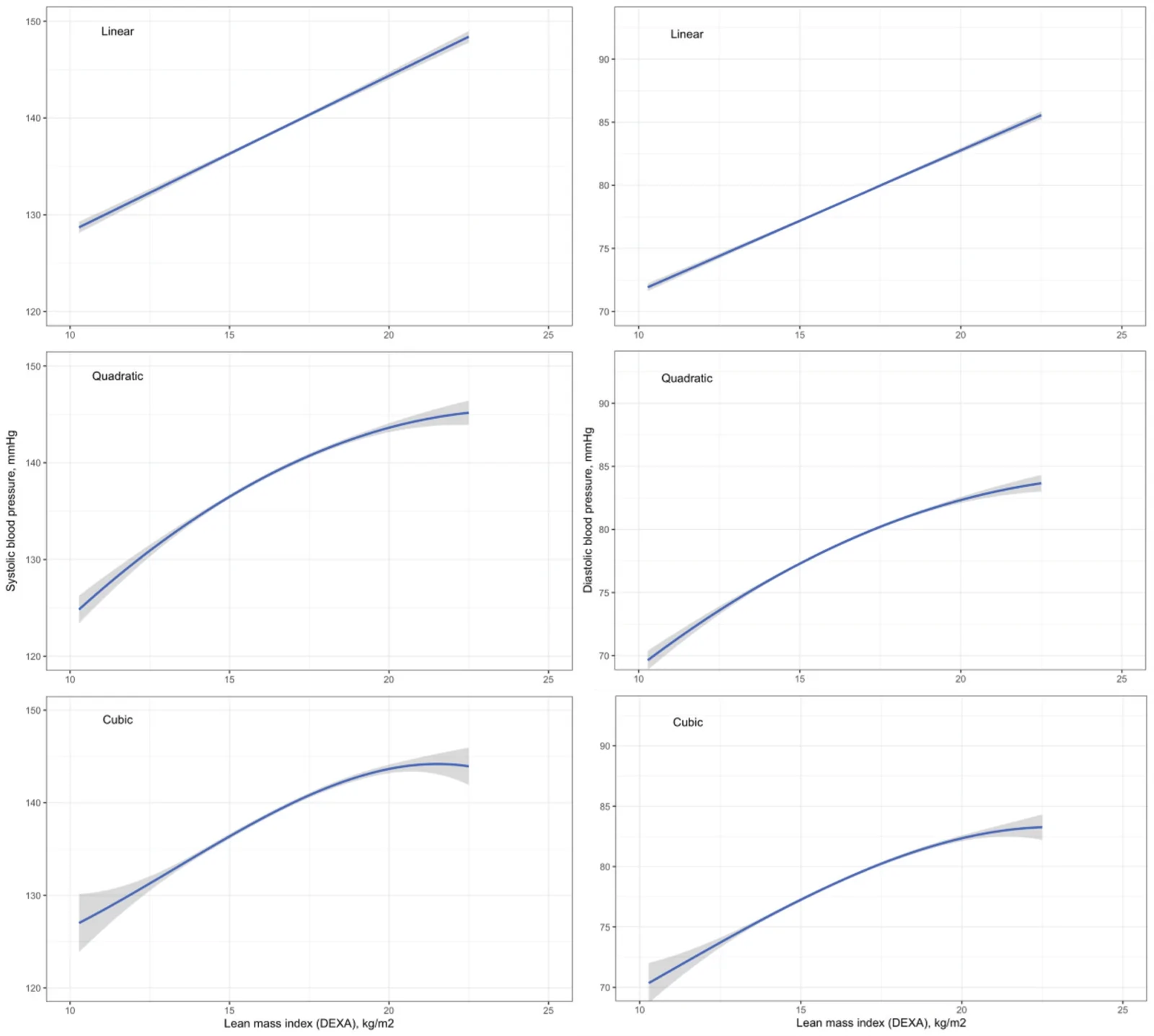
Relationship between dual-energy X-ray absorptiometry muscle mass metrics and blood pressure (baseline cross-sectional data). The left panels show SBP effects, the right panels show DBP effects (linear, quadratic, and cubic). Based on values excluding outliers.

**FIGURE 5:**
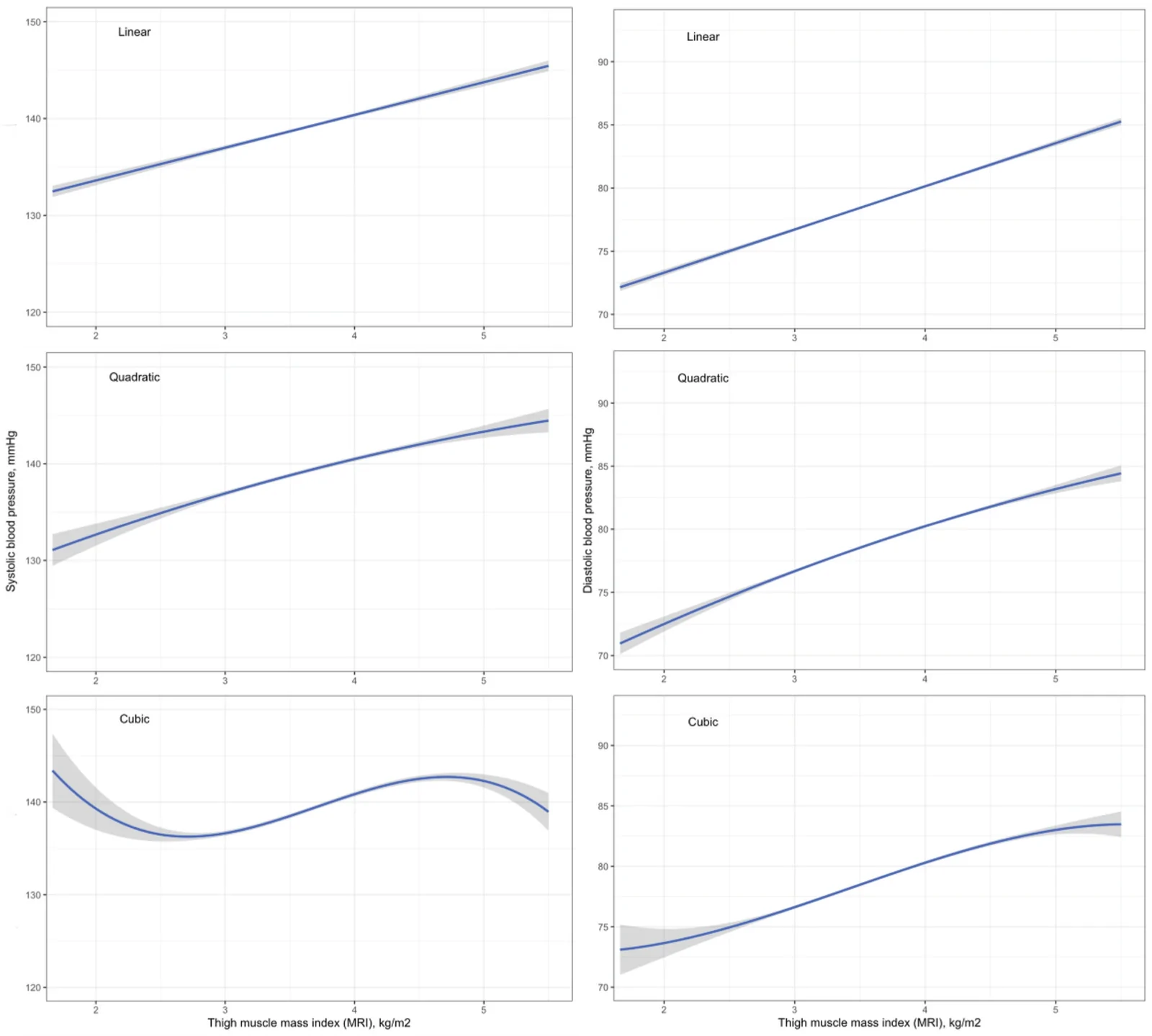
Relationship between MRI muscle mass metrics and blood pressure (baseline cross-sectional data). The left panels show DBP effects, the right panels show DBP effects (linear, quadratic, and cubic). Based on values excluding outliers.

On cross-sectional univariable and multivariable regression, higher values of all study muscle mass metrics were associated with higher SBP (Table 3 and Figs. 3–5). On multivariable analysis (per 1 kg/m^2^ increase) MMI was independently associated with 0.67 (95% CI 0.63–0.71) mmHg increase in SBP, LMI with 0.82 (95% CI 0.68–0.97) mmHg increase in SBP, TMMI with 4.7 (95% CI 4.3–5.1) mmHg increase in SBP (*P* < 0.001 for all, Table 3). FMI increase was also associated with higher SBP (per 1 kg/m^2^ increase): for FMI (BIA) 0.55 (95% CI 0.53–0.57) mmHg, and for FMI (DEXA) 0.73 (95% CI 0.66–0.80) mmHg. A similar pattern was seen in sensitivity analysis (Supplement Table 5).

**TABLE 3:** Regression analysis of relationship of muscle indices and blood pressure using baseline values

	Bioimpedance		DEXA		MRI	
	Beta (95% CI)	*P*	Beta (95% CI)	*P*	Beta (95% CI)	*P*
SBP as outcome						
Univariable	*n* = 482 090		34 097		41 038	
Muscle index^a^	1.4 (1.4–1.4)	<0.001	1.6 (1.5–1.7)	<0.001	3.2 (2.9–3.5)	<0.001
Multivariable	*n* = 474 501		33 416		40,335	
Muscle index^a^	0.67 (0.63–0.71)	<0.001	0.82 (0.68–0.97)	<0.001	4.7 (4.3–5.1)	<0.001
Age	0.69 (0.69–0.70)	<0.001	0.76 (0.73–0.78)	<0.001	0.84 (0.81–0.86)	<0.001
Male sex	4.4 (4.2–4.7)	<0.001	3.6 (3.0–4.2)	<0.001	−0.33 (−0.86 to 0.19)	0.2
Fat mass index	0.55 (0.53–0.57)	<0.001	0.73 (0.66–0.80)	<0.001	-	–
Antihypertensive medications	3.5 (3.4–3.6)	<0.001	2.2 (1.9–3.1)	<0.001	4.0 (3.5–4.6)	<0.001
DBP as outcome						
Univariable	482 092		36 432		43 853	43,095
Muscle index^a^	1.1 (1.0–1.1)	<0.001	1.1 (1.1–1.2)	<0.001	3.5 (3.4–3.7)	<0.001
Multivariable	474 503		35 689		43 095	
Muscle index^a^	0.24 (0.22–0.27)	<0.001	0.31 (0.24–0.30)	<0.001	3.2 (2.9–3.4)	<0.001
Age	0.01 (0.01–0.01)	<0.001	−0.08 (−0.10 to −0.07)	<0.001	−0.04 (−0.05 to −0.03)	<0.001
Male sex	4.6 (4.5–4.7)	<0.001	3.9 (3.6–4.3)	0.001	0.41 (0.13–0.70)	0.004
Fat mass index	0.71 (0.70–0.72)	<0.001	0.63 (0.59–0.67)	<0.001		
Antihypertensive medications	0.73 (0.66–0.80)	<0.001	0.43 (0.13–0.73)	0.006	1.6 (1.3–1.9)	<0.001

a Muscle index refers to muscle mass index for bioimpedance, lean mass index for DEXA, thigh muscle mass index for MRI.

Follow up analyses tested the association between changes in muscle mass metrics and changes in SBP between the follow-up point and baseline. The associations are reported per 1 kg/m^2^ higher value of a muscle mass metric change. On follow-up univariable and multivariable regression, there was a positive association between changes in muscle mass metrics and changes in SBP (Supplement Table 6, Supplement Figure 1). Higher MMI changes during first follow-up of 4.3 (0.9) years were independently associated with 2.2 (95% CI 1.9–2.5) mmHg higher SBP. For the second follow-up of 9.5 (2.2) years, higher MMI change was associated with 2 (95% CI 1.8–2.2) mmHg higher SBP, for the third follow-up of 11.5 (1.2) years higher MMI change was associated with 2.1 (95% CI 1.5–2.6) mmHg higher SBP; higher change in LMI during follow-up of 3.4 (1.7) years was associated with 2.8 (95% CI 1.8–3.8) mmHg higher SBP; higher change in TMMI (i.e. single muscle group rather than whole body muscles) during follow-up of 2.9 (1.3) years was associated with 14 (95% CI 9.3–18) mmHg higher SBP (*P* < 0.001 for all). Larger incremental changes in FMI were also independently associated with higher incremental SBP changes (*P* < 0.001). Similar results were seen in sensitivity analysis (Supplement Table 7).

Along with the linear relationships, we checked for nonlinear relationships but observed weak evidence for nonlinearity, as demonstrated by the corresponding beta coefficients and the little difference in BIC between the nonlinear (quadratic, cubic) and linear models (Figs. 3–5, Supplement Tables 8–9).

### DBP

On cross-sectional univariable and multivariable regression, higher values of all study muscle mass metrics were associated with higher values of DBP (Table 3 and Figs. 3–5). For multivariable analysis (per 1 kg/m^2^ increase), MMI was independently associated with 0.24 (0.22–0.27) mmHg higher values of DBP, LMI with 0.31 (0.24–0.30) mmHg higher values of DBP, TMMI with 3.2 (2.9–3.4) mmHg higher values of DBP (*P* < 0.001 for all, Table 3). FMI increase was also independently associated with higher DBP (per 1 kg/m^2^): for FMI (BMI) 0.71 (0.70–0.72) mmHg, and for FMI (DEXA) 0.63 (0.59–0.67) mmHg. A similar pattern was seen in sensitivity analysis (Supplement Table 5). There were significant interactions between the effect of muscle mass metrics and fat mass metrics (with increasing values of muscle mass, fat mass had less impact on SBP).

Follow-up analyses tested the association between changes in muscle mass metrics and changes in DBP between the follow-up point and baseline. The associations are reported per 1 kg/m^2^ higher value of a muscle mass metric change. On follow-up univariable regression there was a positive association between changes in muscle mass metrics and changes in diastolic BP (Supplement Table 6). For multivariable analysis, higher MMI changes during first follow-up were independently associated with 0.55 (0.39–0.71) mmHg higher values of DBP, MMI during second follow-up with 0.33 (0.22–0.44) mmHg higher values of DBP, LMI with 1.8 (0.76–2.8) mmHg increase in DBP, TMMI (i.e. single muscle group rather than whole body muscles) with 14 (9.2–18) mmHg higher values of DBP (*P* < 0.001 for all). MMI changes during the third follow-up were not significantly associated with changes in DBP, reflecting the observed bi-directional changes in DBP demonstrated above (i.e. prominent reduction of DBP in advanced age). Higher values of FMI changes were independently associated with an increase in DBP for all follow-ups (*P* < 0.001 for all). A similar pattern was seen in sensitivity analysis (Supplement Table 7).

Similar to SBP, nonlinear relationships were also observed, but linear relationships were dominant as demonstrated by corresponding beta coefficients (Figs. 3–5, Supplement Tables 8–9).

### Left ventricular mass index

LVMI was higher in participants with hypertension vs. no hypertension: 48.2 (9.0) vs. 45.1 (8.3) (*P* < 0.001, Table 1). On cross-sectional univariable and multivariable regression, an increase in values of all study muscle mass metrics was associated with higher LVMI (Table 4). For multivariable analysis (per 1 kg/m^2^ increase), MMI was independently associated with 1.3 (1.3–1.4) g/m^2^ LVMI increase; LMI with 1.9 (1.8–2.0) g/m^2^ LVMI increase, TMMI with 4.8 (4.6–4.9) kg/m^2^ LVMI increase (*P* < 0.001 for all). Contrary, higher FMI was associated with lower LVMI: −0.53 (−0.56 to −0.50) mmHg for FMI (BIA) and −0.61 (−0.64 to −0.58) for FMI (DEXA) (*P* < 0.001). A similar pattern was seen in the sensitivity analysis (Supplement Table 10).

**TABLE 4:** Regression analysis of relationship of muscle indices and their changes with left ventricular mass index

	Bioimpedance	DEXA	MRI
	Beta (95% CI)	*P*	Beta (95% CI)	*P*	Beta (95% CI)	*P*
Baseline						
Univariable	n = 37,123		n = 28,710		35,789	
Muscle index^a^	2.0 (2.0–2.1)	<0.001	2.5 (2.4–2.5)	<0.001	8.5 (8.4–8.6)	<0.001
Multivariable	n = 31,500		n = 25,582		30,346	
Muscle index^a^	1.3 (1.3–1.4)	<0.001	1.9 (1.8–2.0)	<0.001	4.8 (4.6–4.9)	<0.001
Age	-0.13 (-0.14--0.12)	<0.001	-0.12 (-0.13--0.11)	<0.001	-0.09 (-0.10--0.08)	<0.001
Male sex	4.1 (3.8–4.4)	<0.001	3.4 (3.2–3.7)	<0.001	5.8 (5.6–6.0)	<0.001
Fat mass index	-0.53 (-0.56--0.50)	<0.001	-0.61 (-0.64--0.58)	<0.001	-	-
SBP	0.15 (0.14–0.15)	<0.001	0.15 (0.14–0.15)	<0.001	0.15 (0.14–0.15)	<0.001
DBP	-0.08 (-0.09--0.07)	<0.001	-0.08 (-0.09--0.07)	<0.001	-0.10 (-0.11--0.10)	<0.001
Follow-up (duration, years)	3.2 (1.6)		3.4 (1.7)		2.9 (1.3)	
Univariable	828		577		672	
Muscle index^a^	0.19 (-0.14–0.52)	0.3	1.4 (0.90–1.8)	<0.001	3.2 (1.5–4.9)	<0.001
Multivariable	673		498		536	
Muscle index change^a^	0.47 (0.07–0.87)	0.020	1.5 (1.0–2.0)	<0.001	3.9 (1.9–5.8)	<0.001
Age	-0.01 (-0.05–0.02)	0.4	-0.01 (-0.05–0.03)	0.5	0.00 (-0.04–0.04)	>0.9
Male sex	-0.25 (-0.75–0.25)	0.3	-0.27 (-0.85–0.31)	0.4	-0.01 (-0.56–0.55)	>0.9
Fat mass index change	-0.35 (-0.57--0.13)	0.002	-0.53 (-0.75--0.31)	<0.001	-	-
SBP change	0.03 (0.00–0.05)	0.018	0.02 (0.00–0.05)	0.048	0.02 (0.00–0.05)	0.049
DBP change	0.02 (-0.02–0.06)	0.3	0.02 (-0.02–0.07)	0.3	0.02 (-0.02–0.06)	0.3

a Muscle index refers to muscle mass index for bioimpedance, lean mass index for DEXA, thigh muscle mass index for magnetic resonance imaging.

Follow-up analyses tested the association between changes in muscle mass metrics and changes in LVMI between the follow-up point and baseline. The associations are reported per 1 kg/m^2^ higher value of a muscle mass metric change. Using univariable regression, increases in LMI and TMMI were associated with higher LVMI BP (Table 4). Using multivariable analysis, higher MMI changes were independently associated with 0.47 (0.07–0.87) g/m^2^ LVMI increase (*P* = 0.02), LMI with 1.5 (1.0–2.0) g/m^2^ LVMI increase (*P* < 0.001), and TMMI with 3.9 (1.9–5.8) g/m^2^ LVMI increase (*P* < 0.001). Contrary, FMI increase was associated with a reduction in LVMI: −0.35 (−0.57 to −0.13) mmHg for FMI (BIA) (*P* = 0.002) and −0.53 (−0.75 to −0.31) for FMI (DEXA) (*P* < 0.001). The above associations were independent of BP values and their changes during follow-up.

## DISCUSSION

This study, for the first time, shows high SMM as a phenotype associated higher raised SBP and DBP and for increased LVMI – a marker of hypertension-related organ damage. The effects were consistent across the three major body composition assessment methods (BIA, DEXA and MRI) and were observed in both cross-sectional and long-term longitudinal analyses. The effect size was also consistent across the three methods, given that thigh muscle mass accounts for approximately 20% of SMM. The effects of muscle mass were independent of body adiposity. The effect of SMM on SBP was predominantly linear and independent of the FMI. The observation suggests that BMI may be inadequate for assessing hypertension risk related to body composition. Differential body composition assessment may benefit future individualized risk profiling for hypertension. It is important to note that longitudinal analyses of the association with DBP must account for the age-related decline in DBP, especially for longer term follow-up. In contrast, cross-sectional analyses were based on single-point DBP values at recruitment, which excluded individuals of advanced age. Results do not provide sufficient evidence to conclude that body composition patterns are associated with BP regulation, and it could be considered a relevant physiological marker.

The longitudinal study found that larger changes in muscle mass, but not fat mass, were independently associated with an increase in LVMI. Of note, the cardiac effects of increased skeletal mass were independent of BP values and their changes during follow-up. Higher LVMI is associated with increased mortality and nonfatal cardiovascular outcomes [8]. In the LIFE trial, in hypertensive patients with left ventricular hypertrophy, better cardiovascular outcomes and mortality in physically active patients were independent of left ventricular mass [9].

BP values are a well established surrogate risk factor for cardiovascular events, even though body composition is not part of hypertension-related risk assessment at present. Some effects of SMM on cardiovascular mobility have already been explored using UK Biobank data. In men, limb muscle mass was associated with a higher cardiovascular disease incidence (hazard ratio per 1 SD 1.07; 95% CI 1.06–1.09) with a curvilinear association in women [10]. A detailed analysis of the effects of SMM on cardiovascular outcomes needs careful consideration of other risk factors, medications and the timing of cardiovascular events, which were outside the scope of this work. The above study suggested J-shaped associations between SMM and overall mortality, which was different from the linear relationship between muscle mass, BP and LVMI in our study [10]. This indicates that the overall health effects of muscle mass are pleiotropic and can be both detrimental and protective, which would need further exploration.

Higher BP and LVMI being associated with higher muscle mass may reflect excessive physical activity, linking associated BP changes with the risk of cardiovascular disease. Of interest, a study showed that 19.2% of football athletes had hypertension, and 61.9% had prehypertension, a high proportion considering the mean age of only 18.6 ± 0.7 years [11]. Skeletal muscle receive 25% of cardiac output at rest and because it is a dynamic tissue, the blood flow could change (increase) remarkably during exercise thus putting more strain on the heart [12]. However, our findings can also be attributed to the increase in muscle mass in response to an increase in fat mass. A noted association of adiposity with LVMI after adjustment for SMM does not imply benefit of higher adiposity. This may be due to the nature of interactions of adiposity and SMM. In a population without sarcopenic frailty, higher adiposity likely leads to an increase in SMM to maintain mobility with the added weight. On the contrary, a proactive increase in SMM (e.g. due to exercise) is more likely to be associated with lower adiposity (relative and/or absolute). Further research is needed to explore the association.

Recent data challenge the BP-lowering effect of increasing activity. A UK randomized clinical trial with open community-based recruitment of 203 physically inactive 18–35-year-old individuals with awake BP 115/75–159/99 mmHg and BMI less than 35 kg/m^2^ 16-weeks aerobic exercise training with motivational coaching to maintain physical activity compared to individuals signposted to educational materials showed no intervention effect of at the end of intervention and 52-weeks follow-up, despite an increase in peak oxygen uptake and peak wattage [13]. A large-scale pairwise and network meta-analysis of randomized controlled trials with various exercise training modes showed an overall improvement in resting BP. Still, it did not consider the impact of changes in muscle mass on the direction and magnitude of BP effects [14]. Together, the data call for a better understanding of the effects of exercise on BP and a balanced approach for recommendations to increase exercise levels.

Cardiomyocytes and skeletal myofibers share a similar basic cellular architecture, both being striated muscle cells. Both cell types are composed of sarcomeres arranged longitudinally in groups to optimize force generation and transmission, and their response to systemic physiological and pathophysiological changes shares common pathways. These include TGF-β signalling, which is broadly implicated in cardiovascular remodelling and health [15].

Assessment of cardiovascular outcomes was outside the scope of this analysis for several reasons. The study did not consider the effects of comorbidities and medications; both could change during the study follow-up. Also, the study did not consider the effects of different types of physical activity and diet. This limitation must be addressed in analyses of interactions between muscle mass, physical activity and BP. A causal effect of changes in muscle mass on changes in BP cannot be reliably established from the study design. The study did not explore the correlation between SMM and cardiac and noncardiac health outcomes. While three methods of SMM evaluation were used in the study, none directly measure SMM; they merely estimate it based on different physicobiological properties of tissues using mathematical modelling (e.g. based on the area of specific muscle groups). The findings are limited by a selective population of the UK Biobank aged 40–69 years at the time of recruitment, with 94% of participants being of white ethnic background with only 5.5% response rate for participation in the study. This limits the generalizability of findings to more diverse or younger populations and potential for bias must be considered. Further studies on populations other than the UK white ethnic background groups need to be conducted to assess generalizability of the findings.

In conclusion, higher SMM is associated with higher BP and LVMI, independently of adiposity. The findings suggest that increased muscularity may reflect unfavourable body remodelling, irrespective of adiposity. Further research is needed to establish whether the observed association is causal and to identify the optimal exercise modes for optimal BP and better health outcomes.

## ACKNOWLEDGEMENTS

This research has been conducted using the UK Biobank Resource under Application Number 54078.

Funding: University of Liverpool internal funding for UK Biobank access.

Data availability: data can be obtained in accordance with UK Biobank Policies https://www.ukbiobank.ac.uk,

### Conflicts of interest

There are no conflicts of interest,

## Supplementary Material

Supplemental Digital Content
