# Supplementary material for: Higher skeletal muscle mass is associated with higher blood pressure and left ventricular mass
Source: J Hypertens. 2026 Jun 20;44(9):1594–604. doi: 10.1097/HJH.0000000000004375 (PMC13566400; doi:10.1097/HJH.0000000000004375)
Supplement: Supplementary file 1 [file jhype-44-1594-s001.docx]

**Supplement Table 1. Rates of hypertension, BP-lowering drug use and raised blood pressure**

|  | Hypertension | | No hypertension | |
| --- | --- | --- | --- | --- |
| n total | 131,476 | | 361,022 | |
| *unknown* | *1,609 (0.01%)* | | *5,458 (0.02%)* | |
|  | No BP-lowering  drugs | On BP-lowering  drugs | No BP-lowering  drugs | On BP-lowering  drugs |
| n | 38,732 | 91,135 | 347,479 | 8,085 |
| Raised systolic BP, n (%) | 25,936 (70%) | 55,009 (62%) | 119,381 (34%) | 3,002 (37%) |
| *unknown* | *1,415 (4%)* | *1,901 (2%)* | *2,989 (1%)* | *76 (1%)* |
| Raised diastolic BP, n (%) | 18,132 (48%) | 27,272 (30%) | 60,781 (18%) | 1,012 (13%) |
| *unknown* | *1,059 (3%)* | *1,000 (1%)* | *2,241 (1%)* | *78 (1%)* |

**Supplement Table 2. Baseline characteristics excluding outliers (sensitivity analysis)**

| **Characteristic** | **Overall** | **No hypertension** | **Hypertension** |
| --- | --- | --- | --- |
| BIA |  |  |  |
| n | 445,788 | 333,888 | 111,900 |
| Age, years | 56 (8) | 55 (8) | 60 (7)^*^ |
| Male sex, n (%) | 204,113 (46%) | 145,187 (43%) | 58,926 (53%)^*^ |
| White ethnicity, n (%) | 423,099 (95%) | 317,582 (95%) | 105,517 (94%)^*^ |
| Smoking, n (%) | 46,351 (10%) | 35,839 (11%) | 10,512 (9.4%)^*^ |
| Diabetes, n (%) | 20,074 (4.5%) | 7,908 (2.4%) | 12,166 (11%)^*^ |
| Myocardial infarction, n (%) | 9,105 (2.0%) | 4,551 (1.4%) | 4,554 (4.1%)^*^ |
| BP-lowering medications, n (%) | 85,348 (19%) | 7,111 (2.1%) | 78,237 (70%)^*^ |
| Cholesterol-lowering  medications, n (%) | 71,386 (16%) | 29,486 (8.8%) | 41,900 (37%)^*^ |
| Systolic BP, mm Hg | 137 (18) | 134 (17) | 146 (16)^*^ |
| Diastolic BP, mm Hg | 82 (10) | 80 (9) | 86 (10)^*^ |
| Body mass index, kg/m2 | 26.9 (3.9) | 26.3 (3.8) | 28.4 (4.0)^*^ |
| Muscle mass index, | 17.5 (2.4) | 17.3 (2.4) | 18.2 (2.5)^*^ |
| Fat mass index, kg/m2 | 8.4 (3.0) | 8.2 (2.9) | 9.3 (3.1)^*^ |
| DEXA |  |  |  |
| n | 32,358 | 26,037 | 6,321^*^ |
| Age, years | 64 (8) | 63 (8) | 67 (7)^*^ |
| Male sex, n (%) | 15,907 (49%) | 12,135 (47%) | 3,772 (60%)^*^ |
| White ethnicity, n (%) | 31,282 (97%) | 25,188 (97%) | 6,094 (96%)^**^ |
| Systolic BP, mm Hg | 138 (18) | 137 (18) | 146 (16)^*^ |
| Diastolic BP, mm Hg | 78 (10) | 78 (10) | 81 (10)^*^ |
| Lean mass index, | 16.3 (2.0) | 16.1 (2.0) | 16.9 (2.1)^*^ |
| Fat mass index, kg/m2 | 8.7 (2.9) | 8.5 (2.9) | 9.6 (3.0)^*^ |
| MRI |  |  |  |
| n | 40,059 | 32,174 | 7,885^*^ |
| Age, years | 64 (8) | 63 (8) | 67 (7)^*^ |
| Male sex, n (%) | 19,118 (48%) | 14,600 (45%) | 4,518 (57%)^*^ |
| White ethnicity, n (%) | 38,877 (97%) | 31,233 (97%) | 7,644 (97%)^**^ |
| Systolic BP, mm Hg | 139 (18) | 137 (18) | 147 (17)^*^ |
| Diastolic BP, mm Hg | 79 (10) | 78 (10) | 81 (10)^*^ |
| Thigh muscle mass index, kg/m2, | 3.6 (0.6) | 3.6 (0.6) | 3.7 (0.6)^*^ |

^*^p<0.001 vs no hypertension; ^**^ p>0.05 vs no hypertension

**Supplement Table 3. Metrics of body composition, blood pressure and LVMI during study visits excluding outliers (sensitivity analysis)**

|  | **Study visit 1** | **Study visit 2** | **Study visit 3** | **Study visit 4** |
| --- | --- | --- | --- | --- |
| BIA |  |  |  |  |
| n | 445,788 | 19,051 | 51,465 | 5,044 |
| Follow up, years | - | 4.3 (0.9) | 9.5 (2.2) | 11.5 (1.2) |
| Age, years | 56 (8) | 61 (7) | 64 (8) | 64 (7) |
| Males, % | 46% | 49% | 48% | 48% |
| MMI, kg/m^2^ | 17.5 (2.4) | 17.3 (2.3) | 17.2 (2.4) | 17.1 (2.3) |
| FMI, kg/m^2^ | 8.4 (3.0) | 8.3 (3.0) | 8.5 (3.3) | 8.4 (3.3) |
| SBP, mm Hg | 137 (18) | 138 (17) | 139 (18) | 140 (18) |
| DBP, mm Hg | 82 (10) | 80 (9) | 79 (10) | 80 (10) |
| LVMI, n |  |  | 36,044 | 817 |
| LVMI follow up, years |  |  | - | 3.2 (1.6) |
| LVMI, g/m^2^ |  |  | 45.3 (7.8) | 45.7 (7.9) |
| DEXA |  |  |  |  |
| n |  |  | 32,358 | 3,103 |
| Follow up, years |  |  |  | 11.5 (1.3) |
| Age, years |  |  | 64 (8) | 64 (7) |
| Males, % |  |  | 49% | 49% |
| LMI, kg/m^2^ |  |  | 16.3 (2.0) | 16.3 (2.0) |
| FMI, kg/m^2^ |  |  | 8.7 (2.9) | 8.6 (3.0) |
| SBP, mm Hg |  |  | 138 (18) | 139 (18) |
| DBP, mm Hg |  |  | 78 (10) | 79 (10) |
| LVMI, n |  |  | 24,090 | 564 |
| LVMI follow up, years |  |  | - | 3.4 (1.7) |
| LVMI, g/m^2^ |  |  | 45.4 (7.8) | 45.6 (7.6) |
| MRI |  |  |  |  |
| n |  |  | 40,059 | 3,465 |
| Follow up, years |  |  |  | 11.2 (1.1) |
| Age, years |  |  | 64 (8) | 64 (7) |
| Males, % |  |  | 48% | 49% |
| TMMI, kg/m^2^ |  |  | 3.6 (0.6) | 3.6 (0.6) |
| SBP, mm Hg |  |  | 139 (18) | 140 (17) |
| DBP, mm Hg |  |  | 79 (10) | 80 (10) |
| LVMI, n |  |  | 29,348 | 657 |
| LVMI follow up, years |  |  |  | 2.9 (1.3) |
| LVMI, g/m^2^ |  |  | 45.2 (7.8) | 45.6 (7.6) |

**Supplement Table 4. Age-related changes in body composition and blood pressure**

| **Age, years** | **40-44** | **45-49** | **50-54** | **55-59** | **60-64** | **65-69** | **70-74** | **75-80** |
| --- | --- | --- | --- | --- | --- | --- | --- | --- |
| n | | | | | | | | |
| *men* | 23,187 | 29,590 | 35,257 | 43,094 | 59,074 | 50,791 | 7,171 | 2,377 |
| *women* | 26,914 | 37,386 | 46,353 | 55,408 | 71,648 | 52,854 | 6,128 | 1,657 |
| Body mass index, kg/m2 | | | | | | | | |
| *men* | 27.5 (4.2) | 27.7 (4.3) | 27.9 (4.4) | 27.9 (4.3) | 27.8 (4.1) | 27.7 (3.9) | 27.0 (3.8) | 26.4 (3.5) |
| *women* | 26.3 (5.4) | 26.6 (5.4) | 27.0 (5.3) | 27.1 (5.2) | 27.2 (5.0) | 27.2 (4.7) | 26.4 (4.5) | 25.7 (4.1) |
| Muscle mass index, kg/m2 | | | | | | | | |
| *men* | 20.0 (1.9) | 19.9 (1.9) | 19.8 (1.9) | 19.7 (1.9) | 19.5 (1.8) | 19.3 (1.7) | 18.8 (1.7) | 18.4 (1.6) |
| *women* | 16.1 (1.6) | 16.1 (1.7) | 16.0 (1.6) | 16.0 (1.6) | 15.9 (1.6) | 15.9 (1.5) | 15.6 (1.5) | 15.4 (1.4) |
| Muscle mass percentage, % | | | | | | | | |
| *men* | 73.2 (5.4) | 72.6 (5.4) | 71.8 (5.5) | 71.3 (5.5) | 70.8 (5.4) | 70.3 (5.3) | 70.3 (5.3) | 70.3 (5.3) |
| *women* | 62.2 (7.0) | 61.6 (6.9) | 60.6 (6.7) | 60.0 (6.5) | 59.6 (6.2) | 59.3 (6.1) | 60.0 (6.0) | 60.6 (6.0) |
| Fat mass index, kg/m2 | | | | | | | | |
| *men* | 6.6 (2.6) | 6.9 (2.6) | 7.1 (2.7) | 7.3 (2.7) | 7.4 (2.6) | 7.5 (2.5) | 7.3 (2.4) | 7.1 (2.3) |
| *women* | 9.4 (3.9) | 9.7 (3.9) | 10.1 (3.9) | 10.3 (3.8) | 10.4 (3.6) | 10.4 (3.5) | 10.0 (3.3) | 9.5 (3.0) |
| Fat mass percentage, % | | | | | | | | |
| *men* | 23.3 (5.6) | 24.0 (5.7) | 24.8 (5.7) | 25.4 (5.7) | 25.9 (5.6) | 26.5 (5.5) | 26.5 (5.5) | 26.5 (5.6) |
| *women* | 34.5 (7.3) | 35.1 (7.2) | 36.2 (7.0) | 36.9 (6.8) | 37.3 (6.5) | 37.5 (6.3) | 36.9 (6.3) | 36.3 (6.3) |
| Systolic BP, mm Hg | | | | | | | | |
| *men* | 133 (14) | 135 (15) | 138 (16) | 141 (17) | 144 (18) | 146 (18) | 147 (18) | 150 (19) |
| *women* | 123 (15) | 127 (16) | 131 (17) | 135 (18) | 140 (19) | 144 (19) | 146 (20) | 151 (21) |
| Diastolic BP, mm Hg | | | | | | | | |
| *men* | 83 (10) | 84 (10) | 85 (10) | 85 (10) | 84 (10) | 83 (10) | 80 (10) | 78 (10) |
| *women* | 78 (10) | 80 (10) | 81 (10) | 81 (10) | 81 (10) | 80 (10) | 78 (10) | 77 (11) |

**Supplement Table 5. Regression analysis of relationship of muscle indices and blood pressure using baseline values excluding outliers (sensitivity analysis)**

|  | **Bioimpedance** |  | **DEXA** |  | **MRI** |  |
| --- | --- | --- | --- | --- | --- | --- |
|  | Beta (95% CI) | p | Beta (95% CI) | p | Beta (95% CI) | p |
| **Systolic blood pressure as outcome** | 475,149 |  | 33,501 |  | 40,348 |  |
| Univariable |  |  |  |  |  |  |
| Muscle index* | 1.4 (1.4-1.4) | <0.001 | 1.6 (1.5-1.7) | <0.001 | 3.4 (3.1-3.7) | <0.001 |
| Multivariable | 454,272 |  | 31,958 |  | 39,653 |  |
| Muscle index* | 0.64 (0.60-0.68) | <0.001 | 0.85 (0.71-0.99) | <0.001 | 4.5 (4.1-4.9) | <0.001 |
| Age | 0.65 (0.64-0.65) | <0.001 | 0.70 (0.68-0.73) | <0.001 | 0.78 (0.75-0.80) | <0.001 |
| Male sex | 4.7 (4.5-4.9) | <0.001 | 3.8 (3.2-4.4) | <0.001 | -0.07 (-0.57-0.43) | 0.8 |
| Fat mass index | 0.66 (0.63-0.68) | <0.001 | 0.81 (0.73-0.88) | <0.001 |  |  |
| Antihypertensive medications | 3.6 (3.5-3.8) | <0.001 | 2.3 (1.7-2.8) | <0.001 | 3.7 (3.2-4.2) | <0.001 |
| **Diastolic blood pressure as outcome** |  |  |  |  |  |  |
| Univariable | 477,124 |  | 36,062 |  | 43,485 |  |
| Muscle index* | 1.0 (1.0-1.0) | <0.001 | 1.1 (1.1-1.2) | <0.001 | 3.4 (3.3-3.6) | <0.001 |
| Multivariable | 456,257 |  | 34,376 |  | 42,732 |  |
| Muscle index* | 0.25 (0.23-0.27) | <0.001 | 0.38 (0.30-0.46) | <0.001 | 3.1 (2.9-3.3) | <0.001 |
| Age | 0.02 (0.01-0.02) | <0.001 | -0.08 (-0.09- -0.06) | <0.001 | -0.04 (-0.05- -0.02) | <0.001 |
| Male sex | 4.5 (4.4-4.7) | <0.001 | 3.7 (3.4-4.0) | <0.001 | 0.33 (0.06-0.61) | 0.019 |
| Fat mass index | 0.78 (0.76-0.79) | <0.001 | 0.68 (0.64-0.72) | <0.001 |  |  |
| Antihypertensive medications | 0.88 (0.81-0.95) | <0.001 | 0.48 (0.18-0.79) | 0.002 | 1.5 (1.2-1.8) | <0.001 |

* Muscle index refers to muscle mass index for bioimpedance, lean mass index for DEXA, thigh muscle mass index for magnetic resonance imaging

**Supplement Table 6. Regression analysis of relationship of changes in muscle indices and blood pressure during follow up**

|  | **Bioimpedance:**  **Follow up 1** | | **Bioimpedance:**  **Follow up 2** | | **Bioimpedance:**  **Follow up 3** | | **DEXA** | | **MRI** | |
| --- | --- | --- | --- | --- | --- | --- | --- | --- | --- | --- |
|  | Beta (95% CI) | p | Beta (95% CI) | p | Beta (95% CI) | p | Beta (95% CI) | p | Beta (95% CI) | p |
| Follow up, years | 4.3 (0.9) |  | 9.5 (2.2) |  | 11.5 (1.2) |  | 3.4 (1.7) |  | 2.9 (1.3) |  |
| **Systolic blood pressure as outcome** | | | | | | | | | | |
| Univariable | 19,474 |  | 43,913 |  | 4,763 |  | 2,396 |  | 2,471 |  |
| Muscle index change* | 2.2  (1.9-2.5) | <0.001 | 2.0  (1.8-2.2) | <0.001 | 2.1  (1.5-2.6) | <0.001 | 2.8  (1.8-3.8) | <0.001 | 14  (9.3-18) | <0.001 |
| Multivariable * | 19,435 |  | 43,824 |  | 4,755 |  | 2,396 |  | 2,471 |  |
| Muscle index change* | 1.4  (1.1-1.7) | <0.001 | 0.97  (0.76-1.2) | <0.001 | 1.0  (0.45-1.6) | <0.001 | 1.8  (0.76-2.8) | <0.001 | 14  (9.2-18) | <0.001 |
| Age | 0.01  (-0.01-0.04) | 0.3 | 0.06  (0.04-0.08) | <0.001 | 0.15  (0.09-0.22) | <0.001 | 0.13  (0.05-0.20) | 0.002 | 0.12  (0.04-0.20) | 0.003 |
| Male sex | -2.9  (-3.3--2.4) | <0.001 | -1.9  (-2.2--1.6) | <0.001 | -2.9  (-3.8--1.9) | <0.001 | -2.4  (-3.6--1.2) | <0.001 | -2.5  (-3.7--1.4) | <0.001 |
| Fat mass index change | 1.5  (1.3-1.6) | <0.001 | 1.4  (1.3-1.6) | <0.001 | 1.2  (0.93-1.5) | <0.001 | 1.6  (1.1-2.1) | <0.001 | - | - |
| **Diastolic blood pressure as outcome** | | | | | | | | | | |
| Univariable | 19,474 |  | 46,946 |  | 4,763 |  | 2,396 |  | 2,471 |  |
| Muscle index change* | 1.2  (1.1-1.4) | <0.001 | 1.3  (1.1-1.4) | <0.001 | 1.1  (0.79-1.4) | <0.001 | 2.8  (1.8-3.8) | <0.001 | 14  (9.3-18) | <0.001 |
| Multivariable | 19,435 |  | 46,849 |  | 4,755 |  | 2,396 |  | 2,471 |  |
| Muscle index change* | 0.55  (0.39-0.71) | <0.001 | 0.33  (0.22-0.44) | <0.001 | -0.05  (-0.38-0.29) | 0.8 | 1.8  (0.76-2.8) | <0.001 | 14  (9.2-18) | <0.001 |
| Age | -0.05  (-0.07--0.04) | <0.001 | -0.13  (-0.14--0.12) | <0.001 | -0.14  (-0.17--0.10) | <0.001 | 0.13  (0.05-0.20) | 0.002 | 0.12  (0.04-0.20) | 0.003 |
| Male sex | -1.3  (-1.5--1.0) | <0.001 | -0.58  (-0.75--0.40) | <0.001 | -0.66  (-1.2--0.10) | 0.020 | -2.4  (-3.6--1.2) | <0.001 | -2.5  (-3.7--1.4) | <0.001 |
| Fat mass index change | 1.4  (1.3-1.5) | <0.001 | 1.3  (1.2-1.3) | <0.001 | 1.4  (1.2-1.5) | <0.001 | 1.6  (1.1-2.1) | <0.001 | - | - |

* Muscle index refers to muscle mass index for bioimpedance, lean mass index for DEXA, thigh muscle mass index for magnetic resonance imaging

**Supplement Table 7. Regression analysis of relationship of changes in muscle indices and blood pressure during follow up excluding outliers (sensitivity analysis)**

|  | **Bioimpedance:**  **Follow up 1** | | **Bioimpedance:**  **Follow up 2** | | **Bioimpedance:**  **Follow up 3** | | **DEXA** | | **MRI** | |
| --- | --- | --- | --- | --- | --- | --- | --- | --- | --- | --- |
|  | Beta (95% CI) | p | Beta (95% CI) | p | Beta (95% CI) | p | Beta (95% CI) | p | Beta (95% CI) | p |
| Follow up, years |  |  |  |  |  |  |  |  |  |  |
| **Systolic blood pressure as outcome** | | | | | | | | | | |
| Univariable | 19,063 |  | 42,924 |  | 4,661 |  | 2,335 |  | 2,415 |  |
| Muscle index change* | 2.2  (1.9-2.5) | <0.001 | 1.9  (1.7-2.1) | <0.001 | 1.8  (1.3-2.3) | <0.001 | 3.5  (2.4-4.6) | <0.001 | 14  (9.7-18) | <0.001 |
| Multivariable* | 18,483 |  | 41,779 |  | 4,542 |  | 2,265 |  | 2,415 |  |
| Muscle index change* | 1.5  (1.2-1.8) | <0.001 | 0.90  (0.69-1.1) | <0.001 | 0.87  (0.28-1.5) | 0.004 | 2.9  (1.7-4.0) | <0.001 | 14  (9.5-18) | <0.001 |
| Age | 0.02  (-0.01-0.04) | 0.2 | 0.05  (0.03-0.07) | <0.001 | 0.12  (0.06-0.18) | <0.001 | 0.09  (0.01-0.17) | 0.024 | 0.09  (0.01-0.17) | 0.020 |
| Male sex | -2.8  (-3.2--2.4) | <0.001 | -1.8  (-2.2--1.5) | <0.001 | -3.0  (-4.0--2.1) | <0.001 | -2.3  (-3.5--1.2) | <0.001 | -2.2  (-3.3--1.1) | <0.001 |
| Fat mass index change | 1.6  (1.4-1.7) | <0.001 | 1.5  (1.4-1.6) | <0.001 | 1.4  (1.1-1.7) | <0.001 | 1.7  (1.2-2.2) | <0.001 |  |  |
| **Diastolic blood pressure as outcome** | | | | | | | | | | |
| Univariable | 19,253 |  | 46,324 |  | 4,695 |  | 2,335 |  | 2,415 |  |
| Muscle index change* | 1.2  (1.1-1.4) | <0.001 | 1.3  (1.1-1.4) | <0.001 | 1.2  (0.84-1.5) | <0.001 | 3.5  (2.4-4.6) | <0.001 | 14  (9.7-18) | <0.001 |
| Multivariable | 18,665 |  | 45,096 |  | 4,572 |  | 2,265 |  | 2,415 |  |
| Muscle index change* | 0.54  (0.37-0.71) | <0.001 | 0.36  (0.25-0.48) | <0.001 | 0.04  (-0.30-0.39) | 0.8 | 2.9  (1.7-4.0) | <0.001 | 14  (9.5-18) | <0.001 |
| Age | -0.06  (-0.07--0.04) | <0.001 | -0.13  (-0.14--0.12) | <0.001 | -0.13  (-0.17--0.10) | <0.001 | 0.09  (0.01-0.17) | 0.024 | 0.09  (0.01-0.17) | 0.020 |
| Male sex | -1.3  (-1.5--1.1) | <0.001 | -0.63  (-0.81--0.46) | <0.001 | -0.92  (-1.5--0.37) | <0.001 | -2.3  (-3.5--1.2) | <0.001 | -2.2  (-3.3--1.1) | <0.001 |
| Fat mass index change | 1.5 (1.4-1.6) | <0.001 | 1.3 (1.3-1.4) | <0.001 | 1.4 (1.3-1.6) | <0.001 | 1.7 (1.2-2.2) | <0.001 |  |  |

* Muscle index refers to muscle mass index for bioimpedance, lean mass index for DEXA, thigh muscle mass index for magnetic resonance imaging

**Supplement Table 8. No-linear effects in the cross-sectional regression analyses**

|  | **Systolic blood pressure** | | | **Diastolic blood pressure** | | |
| --- | --- | --- | --- | --- | --- | --- |
|  | Beta (95% CI) | p | BIC | Beta (95% CI) | p | BIC |
| Muscle mass index (BIA) – Univariable (n=482,090 for SBP, n=482,092 for DBP) | | | | | | |
| *Linear* | 2,485 (2,449-2,521) | <0.001 | 4,170,422 | 1,842 (1,823-1,861) | <0.001 | 3,567,338 |
| *Squared* | -526 (-562- -490) | <0.001 | 4,169,608 | -292 (-312- -273) | <0.001 | 3,566,457 |
| *Cubic* | 20 (16-56) | 0.3 | 4,169,620 | 28 (8.6-47) | 0.005 | 3,566,462 |
| Muscle mass index (BIA) – Multivariable (n=474,501 for SBP, n=474,503 for DBP) | | | | | | |
| *Linear* | 1,250 (1,182-1,319) | <0.001 | 4,039,230 | 512 (474-551) | <0.001 | 3,492,480 |
| *Squared* | -289 (-324- -255) | <0.001 | 4,038,971 | -233 (-252- -214) | <0.001 | 3,491,948 |
| *Cubic* | -11 (-45-24) | 0.5 | 4,038,983 | -94 (-113- -75) | <0.001 | 3,491,870 |
| Lean mass index (DEXA) – Univariable (n=34,097 for SBP, n=36,432 for DBP) | | | | | | |
| *Linear* | 615 (579-652) | <0.001 | 296,377 | 458 (439-477) | <0.001 | 269,948 |
| *Squared* | -104 (-140- -67) | <0.001 | 296,357 | -61 (-80- -41) | <0.001 | 269,920 |
| *Cubic* | -89 (-126- -53) | <0.001 | 296,344 | -57 (-76- -38) | <0.001 | 269,897 |
| Lean mass index (DEXA) – Multivariable (n=33,416for SBP, n=35,689 for DBP) | | | | | | |
| *Linear* | 336 (280-392) | <0.001 | 285,932 | 144 (113-174) | <0.001 | 263,222 |
| *Squared* | -22 (-56-13) | 0.2 | 285,941 | -55 (-74- -35) | <0.001 | 263,203 |
| *Cubic* | -67 (-102- -32) | <0.001 | 285,938 | -47 (-66- -28) | <0.001 | 263,191 |
| Thigh muscle mass index (MRI) – Univariable (n=41,038 for SBP, n=43,853 for DBP) | | | | | | |
| *Linear* | 408 (371-445) | <0.001 | 357,869 | 461 (442-481) | <0.001 | 325,333 |
| *Squared* | -49 (-86- -12) | 0.009 | 357,873 | -43 (-63- -24) | <0.001 | 325,324 |
| *Cubic* | -104 (-141- -67) | <0.001 | 357,854 | -30 (-49- -10) | 0.003 | 325,326 |
| Thigh muscle mass index (MRI) – Multivariable (n=40,335 for SBP, n=43,095 for DBP) | | | | | | |
| *Linear* | 589 (535-643) | <0.001 | 346,115 | 423 (393-453) | <0.001 | 319,594 |
| *Squared* | 47 (12-82) | 0.008 | 346,118 | -37 (-57- -18) | <0.001 | 319,592 |
| *Cubic* | -31 (-66-5.3) | 0.095 | 346,126 | -26 (-46- -5.7) | 0.012 | 319,596 |

DBP, diastolic blood pressure; SBP, systolic blood pressure

**Supplement Table 9. No-linear effects in the regression analysis of follow up changes in blood pressure and muscle indices**

|  | **Systolic blood pressure change** | | | **Diastolic blood pressure change** | | |
| --- | --- | --- | --- | --- | --- | --- |
|  | Beta (95% CI) | p | BIC | Beta (95% CI) | p | BIC |
| Muscle mass index (BIA) change: Follow up 1 (n=19,474 for SBP, n=19,474 for DBP) | | | | | | |
| *Linear* | 222 (193-251) | <0.001 | 160086 | 123 (107-139) | <0.001 | 137349 |
| *Squared* | -77 (-106- -48) | <0.001 | 160069 | -52 (-68- -36) | <0.001 | 137319 |
| *Cubic* | 27 (-2.0-56) | 0.069 | 160075 | 32 (16-48) | <0.001 | 137314 |
| Muscle mass index (BIA) change: Follow up 1 (n=19,435 for SBP, n=19,435 for DBP) | | | | | | |
| *Linear* | 146 (116-175) | <0.001 | 159275 | 55 (39-71) | <0.001 | 135838 |
| *Squared* | -31 (-59- -1.8) | 0.038 | 159279 | -8.6 (-24-7.2) | 0.3 | 135846 |
| *Cubic* | 9.6 (-19-38) | 0.5 | 159289 | 16 (0.05-31) | 0.049 | 135852 |
| Muscle mass index (BIA) change: Follow up 2 (n=43,913 for SBP, n=46,946 for DBP) | | | | | | |
| *Linear* | 343 (309-376) | <0.001 | 374108 | 224 (205- 243) | <0.001 | 346398 |
| *Squared* | 21 (-13-54) | 0.2 | 374117 | -50 (-69- -31) | <0.001 | 346382 |
| *Cubic* | -50 (-83- -16) | 0.004 | 374119 | -22 (-41- -3.3) | 0.021 | 346388 |
| Muscle mass index (BIA) change: Follow up 2 (n=43,824 for SBP, n=46,849 for DBP) | | | | | | |
| *Linear* | 168 (132-203) | <0.001 | 372544 | 60 (40-79) | <0.001 | 343031 |
| *Squared* | 28 (-5.6-61) | 0.10 | 372552 | -50 (-68- -31) | <0.001 | 343014 |
| *Cubic* | -36 (-69- -2.3) | 0.036 | 372558 | -11 (-29- 7.7) | 0.3 | 343024 |
| Muscle mass index (BIA) change: Follow up 3 (n=4,763 for SBP, n=4,763 for DBP) | | | | | | |
| *Linear* | 127 (94-160) | <0.001 | 40416 | 68 (49-88) | <0.001 | 35372 |
| *Squared* | 6.3 (-27-39) | 0.7 | 40424 | -14 (-34-5.0) | 0.14 | 35378 |
| *Cubic* | 17 (-16-50) | 0.3 | 40431 | 7.4 (-12-27) | 0.5 | 35386 |
| Muscle mass index (BIA) change: Follow up 3 (n=4,755 for SBP, n=4,755 for DBP) | | | | | | |
| *Linear* | 62 (27-98) | <0.001 | 40275 | -3.5 (-24-17) | 0.7 | 35005 |
| *Squared* | 13 (-20-46) | 0.4 | 40283 | -10 (-29-8.7) | 0.3 | 35012 |
| *Cubic* | 27 (-6.0-59) | 0.11 | 40288 | 23 (3.9-42) | 0.018 | 35015 |
| Lean mass index (DEXA) change (n=2,396 for SBP, n=2,739 for DBP) | | | | | | |
| *Linear* | 83 (54-112) | <0.001 | 19757 | 44 (27-61) | <0.001 | 19566 |
| *Squared* | 19 (-9.9-48) | 0.2 | 19763 | 0.77 (-16-18) | >0.9 | 19574 |
| *Cubic* | -24 (-53-5.5) | 0.11 | 19769 | -20 (-37- -3.1) | 0.020 | 19576 |
| Lean mass index (DEXA) change (n=2,396 for SBP, n=2,739 for DBP) | | | | | | |
| *Linear* | 52 (22-82) | <0.001 | 19718 | 19 (1.5-36) | 0.033 | 19487 |
| *Squared* | 25 (-3.8-54) | 0.089 | 19723 | 7.5 (-9.0-24) | 0.4 | 19494 |
| *Cubic* | -22 (-51-6.8) | 0.13 | 19729 | -22 (-39- -5.9) | 0.008 | 19495 |
| Thigh muscle mass index (MRI) change (n=2,471 for SBP, n=2,471 for DBP) | | | | | | |
| *Linear* | 90 (61-118) | <0.001 | 20278 | 90 (61-118) | <0.001 | 20278 |
| *Squared* | -7.4 (-36-21) | 0.6 | 20286 | -7.4 (-36-21) | 0.6 | 20286 |
| *Cubic* | -35 (-64- -6.7) | 0.015 | 20288 | -35 (-64- -6.7) | 0.015 | 20288 |
| Thigh muscle mass index (MRI) change (n=2,471 for SBP, n=2,471for DBP) | | | | | | |
| *Linear* | 89 (60-118) | <0.001 | 20269 | 89 (60-118) | <0.001 | 20269 |
| *Squared* | 2.9 (-26-32) | 0.8 | 20276 | 2.9 (-26-32) | 0.8 | 20276 |
| *Cubic* | -38 (-66- -9.2) | 0.009 | 20277 | -38 (-66- -9.2) | 0.009 | 20277 |

DBP, diastolic blood pressure; SBP, systolic blood pressure

**Supplement Table 10. Regression analysis of relationship of muscle indices and their changes with left ventricular mass index excluding outliers (sensitivity analysis)**

|  | **Bioimpedance** |  | **DEXA** |  | **MRI** |  |
| --- | --- | --- | --- | --- | --- | --- |
|  | Beta (95% CI) | p | Beta (95% CI) | p | Beta (95% CI) | p |
| **Baseline** |  |  |  |  |  |  |
| Univariable | 36,557 |  | 28,204 |  | 35,226 |  |
| Muscle index* | 1.9 (1.9-1.9) | <0.001 | 2.4 (2.3-2.4) | <0.001 | 8.1 (8.0-8.2) | <0.001 |
| Multivariable | 30,029 |  | 24,150 |  | 29,420 |  |
| Muscle index* | 1.2 (1.1-1.2) | <0.001 | 1.8 (1.7-1.8) | <0.001 | 4.4 (4.2-4.5) | <0.001 |
| Age | -0.12 (-0.12--0.11) | <0.001 | -0.11 (-0.12--0.10) | <0.001 | -0.08 (-0.09--0.07) | <0.001 |
| Male sex | 4.3 (4.1-4.6) | <0.001 | 3.4 (3.2-3.6) | <0.001 | 5.7 (5.5-5.8) | <0.001 |
| Fat mass index | -0.49 (-0.52--0.46) | <0.001 | -0.57 (-0.60--0.55) | <0.001 |  |  |
| Systolic blood pressure | 0.13 (0.13-0.14) | <0.001 | 0.13 (0.13-0.14) | <0.001 | 0.13 (0.13-0.14) | <0.001 |
| Diastolic blood pressure | -0.07 (-0.08--0.06) | <0.001 | -0.07 (-0.08--0.06) | <0.001 | -0.09 (-0.10--0.08) | <0.001 |
| **Follow up (duration, years)** |  |  |  |  |  |  |
| Univariable | 813 |  | 564 |  | 657 |  |
| Muscle index* | 0.21 (-0.12-0.53) | 0.2 | 1.4 (0.92-1.8) | <0.001 | 2.8 (1.2-4.5) | <0.001 |
| Multivariable | 627 |  | 456 |  | 509 |  |
| Muscle index change* | 0.49 (0.08-0.89) | 0.020 | 1.6 (1.1-2.1) | <0.001 | 3.3 (1.4-5.2) | <0.001 |
| Age | -0.03 (-0.06-0.01) | 0.2 | -0.02 (-0.06-0.02) | 0.3 | -0.02 (-0.06-0.02) | 0.3 |
| Male sex | -0.29 (-0.79-0.22) | 0.3 | -0.36 (-0.94-0.21) | 0.2 | 0.03 (-0.51-0.58) | >0.9 |
| Fat mass index change | -0.36 (-0.59--0.14) | 0.001 | -0.57 (-0.81--0.33) | <0.001 |  |  |
| Systolic blood pressure change | 0.01 (-0.01-0.04) | 0.3 | <0.01 (-0.03-0.03) | 0.9 | 0.01 (-0.02-0.03) | 0.5 |
| Diastolic blood pressure change | 0.04 (0.00-0.08) | 0.064 | 0.05 (0.00-0.10) | 0.033 | 0.04 (-0.01-0.08) | 0.091 |

* Muscle index refers to muscle mass index for bioimpedance, lean mass index for DEXA, thigh muscle mass index for magnetic resonance imaging

**Supplement Table 11. Outliers excluded from the sensitivity analysis**

| Parameter | Low values outliers  (n excluded) | High values outliers  (n excluded) |
| --- | --- | --- |
| Systolic BP, mm Hg | ≤86.5 (58) | ≥187.5 (5516) |
| Diastolic BP, mm Hg | ≤53.5 (443) | ≥110.5 (3041) |
| BMI (physical assessment), kg/m2 | ≤15.4756 (76) | ≥38.563 (13,930) |
| BMI (BIA), kg/m2 | ≤15.4 (55) | ≥38.6 (13,630) |
| FMI (BIA), kg/m2 | - | ≥17.22681 (15,052) |
| MMI (BIA), kg/m2 | ≤9.413217 (16) | ≥25.23972 (1761) |
| FMI (DEXA), kg/m2 | 0 (1) | ≥16.98572 (1129) |
| LMI (DEXA), kg/m2 | ≤9.375816 (6) | ≥22.50953 (165) |
| BMI (DEXA), kg/m2 | ≤15.2906 (65) | ≥36.61742 (1244) |
| Thigh MMI (MRI), kg/m2 | ≤1.6634475 (4) | ≥5.499628 (133) |
| LVMI, kg/m2 | ≤21.21.90328 (11) | ≥68.33934 (524) |

* Patients without stroke

**Supplement Figure 1. Relationship between muscle mass metrics and blood pressure (follow up changes)**

Top three panels show systolic BP effects and the lower three panel show diastolic BP effects (linear, quadratic, and cubic). From left to right, first three panels show bioimpedance data for follow ups 1, 2 and 3, the fourth panel shows DEXA, and the right panel shows magnetic resonance imaging. Based on values excluding outliers
